# Belowground Fungal Volatile Exposure Induces Systemic Metabolic Reprogramming in Poplar

**DOI:** 10.1111/ppl.71147

**Published:** 2026-10-09

**Authors:** Peiyuan Zhu, Ina Zimmer, Prasath Balaji Sivaprakasam Padmanaban, Maaria Rosenkranz, Andrea Ghirardo, Jörg‐Peter Schnitzler

**Affiliations:** ^1^ Research Unit Environmental Simulation Helmholtz Munich Neuherberg Germany; ^2^ Institute of Plant Sciences, Ecology and Conservation Biology University of Regensburg Regensburg Germany; ^3^ Institute for Translational Metabolism Research, Helmholtz Diabetes Center, Helmholtz Munich/School of Medicine and Health, Technical University of Munich Munich Germany; ^4^ Ecosystem Physiology, Faculty of Environment and Natural Resources University Freiburg Breisgau Germany

**Keywords:** fungal volatiles, metabolomics, plant‐fungal communication, *Populus*, root‐to‐shoot communication, systemic signalling, volatile organic compounds

## Abstract

Although trees encounter diverse soil‐associated fungi, it is unclear how they adjust their physiology in response to volatile organic compounds (VOCs) from fungi with contrasting ecological strategies before physical contact. Here, we tested whether VOCs emitted by fungi are sufficient to induce systemic, fungus‐dependent metabolic responses in poplar. *Populus* × *canescens* roots were exposed to VOCs from a pathogen (*Heterobasidion annosum*), a saprotroph (*Postia placenta*), or an ectomycorrhizal mutualist (*Laccaria bicolor*) for 6 weeks in a contact‐free pot‐in‐pot system. Untargeted LC–MS metabolomics was used to characterize VOC‐induced metabolic reprogramming in roots and leaves. Fungal VOC exposure alone reconfigured root and leaf metabolomes, with both shared and fungus‐dependent responses despite VOC exposure being restricted to the belowground compartment. Treatment‐associated differences varied between tissues. *H. annosum* was associated with pronounced suppression of high‐confidence features in leaves but a more balanced response in roots. *P. placenta* showed several uniquely upregulated root features together with selected lipid‐associated changes, and *L. bicolor* produced comparatively few unique high‐confidence changes. These results show that poplar can respond systemically and differentially to VOCs from contrasting fungal species before physical contact, while the extent to which these responses can be generalised across fungal lifestyles remains to be established.

## 1 Introduction

Plants develop and function within dense belowground communities, where their roots are continuously exposed to fungi that span pathogenic, saprotrophic, and mutualistic lifestyles. These interactions influence nutrient acquisition, growth, and stress resistance, and are particularly important in forest ecosystems, where long‐lived hosts must process microbial signals over extended periods (Baldrian 2017; van der Heijden et al. 2015; Philippot et al. 2013). Although contact‐dependent recognition, such as pattern‐recognition receptors detecting fungal cell‐wall components and triggering immune signalling, is well described (Doehlemann et al. 2017; Spoel and Dong 2012), the manner in which plants assess fungal identity and interaction context prior to physical contact remains unclear.

Volatile organic compounds (VOCs) offer a potential route for such pre‐contact recognition. VOCs are small, diffusible metabolites that move through soil, air and water, enabling information transfer between spatially separated organisms (Peñuelas et al. 2014; Weisskopf et al. 2021). Fungal volatilomes are chemically diverse and dynamic, with their composition shaped by growth conditions and environmental factors. Reviews have highlighted their ecological roles as mediators of interspecific interactions (Morath et al. 2012; Yin et al. 2025). For example, fungal VOCs can modify plant root architecture and alter plant defence responses (Ditengou et al. 2015; Weisskopf et al. 2021). Fungal VOC blends are shaped by trophic mode and ecological strategy, suggesting that their blend structure may convey information associated with fungal ecology rather than relying on individual compounds alone (Guo et al. 2021; Müller et al. 2013).

The concept of chemical communication displays (CCDs) is an explicitly mixture‐based view, capturing multicomponent bouquets that encode biologically meaningful information in their composition, relative ratios, and covariation among components (Junker et al. 2018). Guo et al. (2021) applied the CCD theory to fungi, demonstrating that VOC patterns can predict trophic mode and lifestyle across taxa, and quantifying ‘phenotypic integration’ (compound covariation) within volatilomes. The CCD theory thus offers a mechanistic framework for how plants could decode ‘who is there’ from volatile bouquets, even when individual components vary with growth stage or substrate.

Experimental evidence supports volatile‐mediated plant‐fungal interactions. Fungal VOCs can alter plant growth, root system architecture, and defence‐related physiology without hyphal contact (Bitas et al. 2013; Moisan et al. 2020; Singh et al. 2021), which aligns with the broader role of microbe‐induced plant volatiles in plant‐microbe ecology (Sharifi and Ryu 2018). Sesquiterpenes emitted by the ectomycorrhizal fungus *Laccaria bicolor* (Maire) P.D. Orton are sufficient to reprogramme host root development in the absence of physical interaction, demonstrating that VOCs can act as targeted developmental signals (Ditengou et al. 2015). However, many studies focus on short‐term responses or individual compounds, leaving unresolved the question of whether plants can reliably discriminate among complex VOC mixtures from fungi with contrasting ecological lifestyles under standardised conditions.

Using a contact‐free pot‐in‐pot system, we recently reported (Zhu et al. 2026) that Grey poplar (*Populus* × *canescens* (Aiton) Sm.) can discriminate VOC cues from three fungi: a pathogenic fungus (*Heterobasidion annosum* (Fr.) Bref.), a saprotrophic fungus (*Postia placenta* (Fr.) Niemelä, K.H. Larss. & Schigel), and an ectomycorrhizal fungus ( *L. bicolor* ). Root‐zone headspace VOC signatures were dominated by sesquiterpenes and remained distinguishable among fungal treatments over time. In contrast, leaf VOC emissions were more diverse and partially converged (Zhu et al. 2026). While these findings demonstrate that poplar responds differently to VOC cues from the three fungi, they do not reveal how these volatile cues are translated into internal, systemic physiological states.

Plant hormone signalling networks, particularly those involving jasmonic acid (JA), ethylene (ET) and salicylic acid (SA), provide a mechanistic context for such state transitions (Spoel and Dong 2012; Wasternack and Hause 2013). Antagonistic interactions typically trigger early JA/ET responses that interact with SA‐associated systemic signalling, whereas mutualistic interactions involve more controlled modulation of these pathways. In the *Populus*‐ *L. bicolor* symbiosis, jasmonate signalling is attenuated by fungal effectors such as MiSSP7, which interfere with JAZ‐mediated regulation, suppressing defence and permitting developmental reprogramming (Daguerre et al. 2020; Felten et al. 2009). This context‐dependent adjustment is evolutionarily significant as ectomycorrhizal fungi have evolved repeatedly from saprotrophic ancestors, accompanied by the extensive remodelling of capacities related to decay and the emergence of traits associated with symbiosis (Kohler et al. 2015; Wu et al. 2022). These signalling differences provide a physiological context for understanding how plants may respond differently to fungal cues. However, whether fungal VOCs alone are sufficient to induce distinct systemic metabolic responses before physical contact remains unclear.

Here, we investigate whether VOCs from the three contrasting fungal species induce distinct systemic metabolic responses in a woody host. We analysed the metabolomes of the roots and leaves of Grey poplar, which were exposed to VOCs from *H. annosum*, *P. placenta* or *L. bicolor* for 6 weeks, using the same contact‐free exposure experiment and plant cohort as in Zhu et al. (2026) for volatile profiling. Integrating endpoint metabolomics with time‐resolved volatilomics and CCD theory (Guo et al. 2021; Junker et al. 2018) allows us to test the hypothesis that fungal VOCs from contrasting fungal species induce distinct root and systemic metabolic responses prior to physical contact.

## 2 Materials and Methods

### 2.1 Plant Material and Growth Conditions

Wild‐type *Populus* × *canescens* (Aiton) Sm. (INRA clone 717‐1B4, Mader et al. 2016) plantlets were used. Plantlets from sterile stock were sectioned and cultured in glass jars with half‐strength Murashige & Skoog medium (Duchefa Biochemie) in a growth chamber at 20/16°C (day/night) with a 10/14 h photoperiod (100 μmol m^−2^ s^−1^ PPFD) for 2 months. Rooted plantlets were transferred to autoclaved substrate (soil:vermiculite:perlite, 3:1:1, v/v/v) and acclimated in a phytochamber at 22/16°C (light/dark), 60/80% relative humidity (light/dark), and a 10/14 h photoperiod (150 μmol m^−2^ s^−1^ PPFD) for 3 months, with plastic covers used initially to maintain high humidity and gradually removed over 2 weeks to acclimate the plantlets to the chamber conditions.

### 2.2 Fungal Material and Culture Conditions

Three fungal strains representing distinct ecological lifestyles were applied: pathogenic *Heterobasidion annosum* (Fr.) Bref. (stock: Karin Pritsch, Helmholtz Munich), ectomycorrhizal *Laccaria bicolor* (Maire) P.D. Orton (S238N), and saprotrophic *Postia placenta* (Fr.) Niemelä, K.H. Larss. & Schigel (stock: K. Pritsch). Cultures were grown in 35 mm × 10 mm dishes (Corning 430588, Corning Inc.) on modified Melin‐Norkrans (MMN) medium [1% (w/v) glucose, 0.25% (w/v) NH_4_‐tartrate, 0.025% (w/v) (NH_4_)_2_SO_4_, 0.05% (w/v) KH_2_PO_4_, 0.015% (w/v) MgSO_4_·7H_2_O, 0.005% (w/v) CaCl_2_·2H_2_O, 0.0025% (w/v) NaCl, 0.01% (w/v) FeCl_3_, 0.001% (w/v) thiamine hydrochloride, 1% (w/v) Gelrite; pH 5.2; Guo et al. 2021]. Fungal cultures were maintained at room temperature in the dark.

### 2.3 Pot‐in‐Pot Exposure System

A custom pot‐in‐pot (PiP) system enabled root exposure to fungal VOCs without physical contact. The PiP components were wrapped in aluminium foil and autoclaved at 121°C for 20 min before use. Upper pots filled with 180 g of autoclaved substrate (soil:vermiculite:perlite, 3:1:1, v/v/v) had perforations in the lower 2 cm (28 holes, 1 mm diameter) to allow gas exchange and were positioned above lower pots containing fungal cultures. Poplar‐present systems were assigned to four lower‐pot treatments: fungus‐free MMN control, *H. annosum*, *L. bicolor* , and *P. placenta* (VOC exposure: *n* = 6 plants per treatment). Trays were rotated every 3 days. Fungal plates were replaced weekly with pre‐colonised cultures (*H. annosum*: 8 days; *L. bicolor* : 28 days; *P. placenta* : 4 days pre‐cultivation), with species‐specific pre‐cultivation periods selected to ensure well‐established surface colonization at the start of each exposure interval, while control systems received fresh fungus‐free MMN plates on the same schedule. VOC exposure lasted 6 weeks. This duration was selected to assess the established endpoint metabolic state resulting from sustained and repeatedly renewed fungal VOC exposure, rather than acute responses immediately following VOC perception. This pot‐in‐pot exposure experiment was the same experimental run used for volatile profiling in Zhu et al. (2026), whereas the present study focuses on the endpoint LC–MS metabolomic analysis of tissues collected from those same plants.

### 2.4 Sample Collection and Metabolite Extraction

Plant harvest for metabolomic analysis was performed at the end of the six‐week VOC exposure period and coincided with the final volatile sampling of the same experimental run reported in Zhu et al. (2026), enabling direct comparison of VOC signatures and metabolome outcomes within the same exposure system. Root and leaf tissues were collected for metabolomic analysis (*n* = 4 per treatment for roots; *n* = 4 per fungal treatment and *n* = 3 for the leaf control). Although six plants were initially established per treatment, LC–MS acquisition constraints and the amount of remaining plant material restricted metabolomic analysis to four biological replicates. One control leaf sample contained insufficient material for extraction, resulting in *n* = 3 for this group. During VOC exposure, we checked weekly to ensure there was no physical contact between roots and fungi. Roots were excavated, washed with deionized water, gently blotted dry to remove residual surface water, and flash‐frozen in liquid nitrogen. The uppermost 2–3 fully expanded leaves were pooled per plant and flash frozen. All samples were stored at −80°C, then freeze‐dried and ground to a fine powder for metabolite extraction, following Bertić et al. (2021) and Sivaprakasam Padmanaban et al. (2025). Fifty mg of tissue powder was extracted with 1 mL ice‐cold methanol/2‐propanol/water (1:1:1, v/v/v), vortexed for 30 s, sonicated for 10 min at 4°C, and centrifuged at 10,000 *g* for 10 min at 4°C. Supernatants were vacuum‐dried and reconstituted in 200 μL of 50% acetonitrile. Quality control (QC) samples were prepared by pooling equal volumes of all study extracts. Pooled QC injections were analysed every five study samples throughout the sequence to monitor analytical stability. Solvent blanks were injected at the beginning and end of the batch to assess background and carryover.

### 2.5 Liquid Chromatography‐Mass Spectrometry (LC–MS) Analysis

Untargeted metabolomics analysis was performed using an ultra‐high performance liquid chromatography (UHPLC/UPLC) system coupled to an ultra‐high resolution (UHR) QqToF mass spectrometer. Metabolite profiling was conducted on an Ultimate 3000RS UPLC (Thermo Fisher Scientific) coupled to a Bruker Impact II QqToF mass spectrometer equipped with an electrospray ionisation (ESI) source. Chromatographic separation was achieved using reversed‐phase LC (RPLC) on an Acquity BEH C18 column (150 × 2.1 mm, 1.7 μm; Waters) maintained at 40°C, with mobile phases of (A) water +0.1% (v/v) formic acid and (B) acetonitrile +0.1% (v/v) formic acid. The injection volume was 5 μL at a flow rate of 0.35 mL min^−1^ (ESI+) and 0.4 mL min^−1^ (ESI−). Mass spectrometric detection was conducted in separate runs in both ESI+ and ESI− modes. Key parameters were: capillary voltage 4.5 kV (ESI+) / 3.5 kV (ESI−), end plate offset −500 V, nebulizer pressure 2.0 bar, drying gas 8.0 L min^−1^ at 200°C. Spectra were acquired over m/z 20–1000 at 8 Hz. Data‐dependent MS/MS was performed on the top 3 precursors per cycle using CID (collision‐induced dissociation).

Raw LC–MS data were processed in MetaboScape 4.0 (Bruker Daltonics) for peak detection, chromatographic alignment, and isotope/adduct grouping. Mass features detected in ESI+ and ESI− modes were processed separately and subsequently merged for downstream analysis. Metabolite annotation was performed based on accurate mass and MS/MS spectral matching following the workflow described by Bertić et al. (2021). Compound identification relied on spectral library searches against databases including KEGG, HMDB, LIPID MAPS, and PlantCyc. Features were reported as MSI Level 2 (putatively annotated compounds) when MS/MS matches achieved a spectral score > 700 with a mass error < 5 ppm. For features without MS/MS matches, elemental formulas were predicted using SmartFormula, and tentative annotations were assigned at MS1 level using 10 mDa tolerance for the precursor mass with in‐house R code. All detected features were additionally classified using multidimensional stoichiometric compound classification (MSCC) (Rivas‐Ubach et al. 2018) based on an assessment of the C/H/O/N/P stoichiometric ratios, enabling functional grouping of unannotated metabolites into biochemical classes (e.g., lipids, secondary metabolites, peptides/amino acids).

### 2.6 Statistical Analysis

Prior to statistical analysis, peak areas were normalized using median normalization to correct for systematic variation among samples. Multivariate analyses on mass feature data were performed in SIMCA 13 (v13.0.3.0, Umetrics) and univariate statistics and visualisation in R v4.3.1 (R Core Team 2023). Before multivariate analyses, mass feature data were log_10_‐transformed, mean‐centred, and Pareto‐scaled. Before supervised modelling, we first performed Principal Component Analysis (PCA), Uniform Manifold Approximation and Projection (UMAP), and Non‐metric Multidimensional Scaling (NMDS; Euclidean distance) (Figure S1). These unsupervised methods were applied to visualize global data structure, identify outliers, and evaluate treatment‐related patterns prior to Orthogonal Partial Least Squares‐Discriminant Analysis (OPLS‐DA). OPLS‐DA was then used to assess metabolic discrimination between treatments in root and leaf tissues separately. Model quality was evaluated using *R* ^2^ *X*, *R* ^2^ *Y*, and *Q* ^2^, with validation by 200 permutation tests. Model significance was assessed by CV‐ANOVA at *p* < 0.05. Discriminant features were first defined using VIP > 1. High‐confidence differential features were defined using VIP > 1, |log_2_FC| > 0.585 (i.e., 1.5‐fold difference between treatments), and FDR‐adjusted *p* < 0.05 (Welch's *t*‐test with Benjamini‐Hochberg correction). For individual metabolite comparisons, data were log_2_‐transformed to improve normality (assessed by the Shapiro–Wilk test) and analysed using Welch's one‐way ANOVA (unequal variances), followed by Games‐Howell post hoc tests (Perez‐Fons et al. 2023); *p‐*values were adjusted using the Benjamini‐Hochberg procedure (FDR), with FDR‐adjusted *p* < 0.05 considered significant. To assess functional pathway activity, a Module Activity Score was calculated by averaging the *Z*‐score normalised abundances of annotated metabolites mapped to a specific KEGG module or functional class. Significant differences in module scores were determined using *t*‐tests with FDR correction. Hierarchical clustering (Euclidean distance) was applied to *Z*‐score normalised differential features. Pearson correlation networks were constructed within each tissue across all samples (roots: *n* = 16; leaves: *n* = 15) using the high‐confidence differential feature set as nodes and analysed using the *igraph* package (Csárdi and Nepusz 2006) in R. Edges represented significant Pearson correlations among features (|*r*| > 0.7, *p* < 0.05), and treatment‐specific responses were overlaid as node attributes (direction of change relative to control and primary responder, defined as the treatment with the largest absolute log_2_FC). Network modules were identified via the Louvain algorithm (Blondel et al. 2008; Rahiminejad et al. 2019) and assigned to functional categories based on MSCC classifications and available structural annotations. Network visualisation employed the Fruchterman‐Reingold force‐directed layout algorithm (Fruchterman and Reingold 1991). To assess whether VOC chemical classes predict metabolome variation, generalised linear models (GLMs) were fitted to link tissue‐specific metabolome PC1 scores with VOC data from Zhu et al. (2026), including aggregated VOC abundances by chemical class and categorical predictors (dominant sesquiterpene/monoterpene class, treatment). Models were evaluated using both logistic regression (binomial family, *χ* ^2^ test) for binarized outcomes and linear regression (F‐test) for continuous outcomes. Raw metabolomics data, processed feature matrices, and custom R scripts are available in the OSF (Open Science Framework) repository (DOI: 10.17605/OSF.IO/7Y4E8; https://osf.io/7y4e8/).

## 3 Results

### 3.1 Fungal VOCs Induce Distinct Metabolic Profiles in Poplar Roots and Leaves

To investigate whether fungal VOCs can induce metabolic reprogramming in the absence of physical contact, we employed a non‐contact pot‐in‐pot system (Figure 1a) to expose *Populus* × *canescens* roots to VOCs emitted by fungi with different ecological lifestyles: the pathogen *Heterobasidion annosum*, the ectomycorrhizal fungus *Laccaria bicolor*, and the saprotroph *Postia placenta*. Untargeted metabolomics detected 949 metabolic features across all the samples (OSF repository), of which 94 were putatively annotated to chemical structures by MS/MS spectral matching. An additional 263 compounds were annotated based on MS1 data, yielding an overall annotation rate of 37.6% (357/949). Unannotated features were classified into functional categories (lipids, secondary metabolites, peptides/amino acids, carbohydrates, and unknowns) using the MSCC method.

**FIGURE 1:**
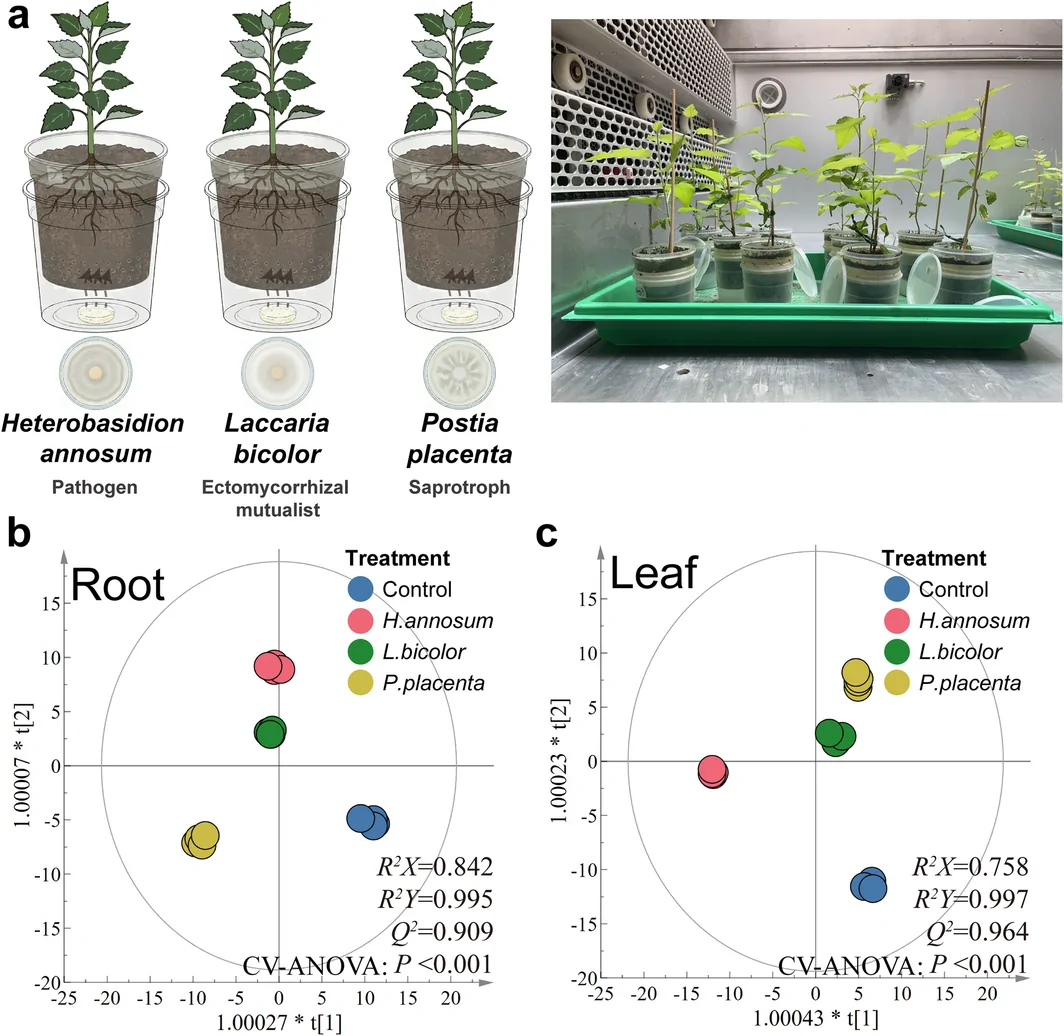
Experimental design and metabolic discrimination of poplar tissues following root exposure to fungal volatile organic compounds (VOCs). (a) Schematic illustration of the pot‐in‐pot (PiP) exposure system, prepared by the authors using Inkscape (left), and a photograph of the experimental setup (right). *Populus* × *canescens* plants were grown in upper pots with perforated bases and positioned above lower pots containing fungal cultures on MMN medium. Three fungi representing distinct ecological lifestyles were used: the pathogen *H. annosum*, the ectomycorrhizal fungus *L. bicolor* , and the saprotroph *P. placenta* . This setup enabled root exposure to fungal VOCs without physical contact (VOC exposure: *n* = 6 plants per treatment). (b, c) orthogonal partial least squares‐discriminant analysis (OPLS‐DA) score plots of root (b) and leaf (c) LC–MS feature profiles after 6 weeks of VOC exposure. Each point represents one biological replicate included in the metabolomics dataset (*n* = 4 per treatment; leaf control *n* = 3). Ellipses indicate the 95% Hotelling's *T* ^2^ confidence ellipse. Model quality parameters (*R* ^2^ *X*, *R* ^2^ *Y*, *Q* ^2^) and cross‐validated ANOVA (CV‐ANOVA) significance are shown.

OPLS‐DA revealed significant separation in root metabolic profiles among all four groups (*R* ^2^ *X* = 0.842, *R* ^2^ *Y* = 0.995, *Q* ^2^ = 0.909; CV‐ANOVA: *p* < 0.001; Figure 1b). All three fungal VOC treatments showed distinct root metabolic profiles relative to the control, with treatment‐specific positioning in the score plot. In leaves, metabolome profiles were similarly discriminated among treatments (*R* ^2^ *X* = 0.758, *R* ^2^ *Y* = 0.997, *Q* ^2^ = 0.964; CV‐ANOVA: *p* < 0.001; Figure 1c). Leaf tissues showed clear treatment‐associated separation despite VOC exposure occurring only belowground, indicating systemic metabolic reprogramming. The relative positioning of the fungal treatments differed between roots and leaves, consistent with tissue‐dependent metabolic responses to fungal VOC exposure.

Principal component analysis (PCA) revealed treatment‐associated clustering of metabolomic profiles in roots and leaves (Figure S1a,b). This organisation was supported by UMAP analyses, which preserve local neighbourhood structure, and by NMDS ordination based on the Euclidean distance of scaled data (Figure S1c–f). Leaves showed clearer separation between treatments than roots in these ordinations, despite VOC exposure being restricted to the belowground compartment. The broadly similar treatment‐associated patterns across PCA, UMAP and NMDS supported overall agreement among complementary unsupervised ordinations prior to supervised discrimination and downstream mass feature selection.

### 3.2 Discriminant Feature Profiles Reveal Shared and Treatment‐Associated Responses

Based on the OPLS‐DA model, mass features with VIP values greater than 1 were selected to examine treatment‐related response patterns, yielding 172 mass features in roots and 113 in leaves (Figure S2). Heatmap analysis revealed tissue‐dependent metabolic changes (Figure S3a,d), with compound classes including carbohydrates, lipids, secondary metabolites, peptides, and amino acids. In roots, all fungal treatments clustered together but showed distinct regulation patterns. In leaves, *H. annosum* showed a unique profile dominated by downregulated mass features. Venn diagrams showed that many mass features were shared across all fungal treatments (21 upregulated and 54 downregulated in roots; Figure S3b,c,e,f), suggesting a common metabolic response to fungal VOCs. Volcano plots further illustrated treatment‐associated distributions of up‐ and downregulated features across the three fungal treatments (Figure S4).

To identify high‐confidence differential features, stricter criteria were applied (VIP > 1, |log_2_FC| > 0.585, FDR‐adjusted *p* < 0.05), resulting in 58 and 63 high‐confidence mass features in roots and leaves, respectively (Figure 2). Under these criteria, the shared response was reduced (from 21 to 8 upregulated and from 54 to 6 downregulated in roots; Figure 2b,c), indicating that many shared mass features had moderate fold changes or high variability. However, treatment‐specific patterns remained clear. *H. annosum* uniquely suppressed 21 leaf mass features, one‐third of all high‐confidence leaf mass features, but showed no unique upregulation in leaves. *P. placenta* retained 11 uniquely upregulated root mass features. *L. bicolor* showed the largest reduction in unique mass features (from 16 to 2 upregulated in roots), suggesting its response overlaps more with other treatments. Van Krevelen diagrams were additionally used to visualise the elemental‐composition space and MSCC classifications of these high‐confidence differential features in roots and leaves (Figure S5).

**FIGURE 2:**
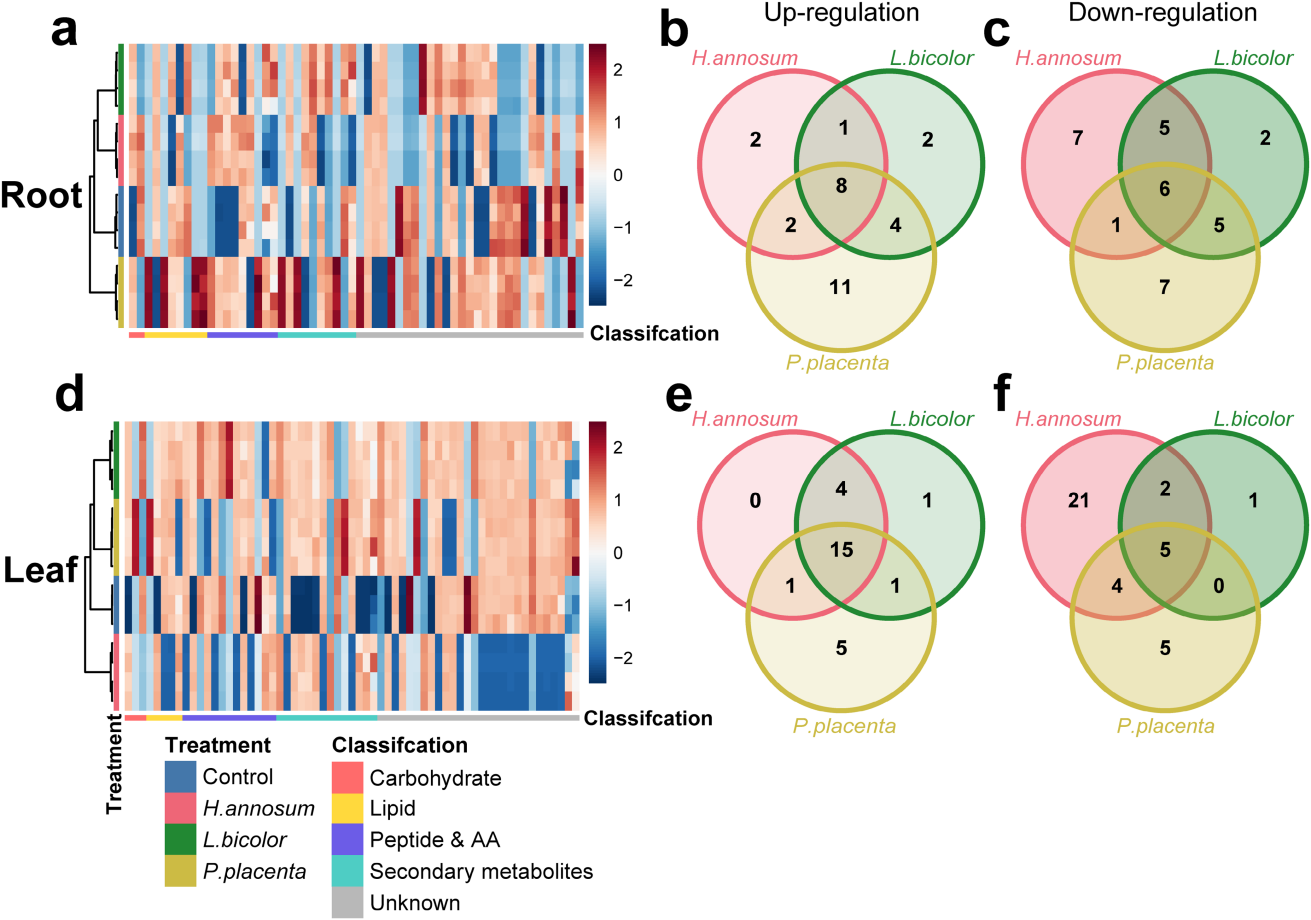
High‐confidence differential feature signatures in poplar tissues following root exposure to fungal VOCs. (a, d) Heatmaps showing *Z*‐score normalised abundance of differential features (VIP > 1, |log_2_FC| > 0.585, FDR‐adjusted *p* < 0.05) in root (a) and leaf (d) tissues. Hierarchical clustering (Euclidean distance, complete linkage) was applied to samples (rows). Colour scale indicates relative abundance (red: elevated; blue: reduced). Left annotation bars indicate treatment groups. Bottom annotation bars show compound classifications based on Multidimensional Stoichiometric Compound Classification (MSCC). (b, c, e, f) Venn diagrams displaying the overlap of upregulated (b, e) and downregulated (c, f) features among treatments in root (b, c) and leaf (e, f) tissues.

### 3.3 Correlation Networks Reveal Tissue‐Dependent Metabolic Co‐Variation

To investigate co‐variation among high‐confidence differential mass features, we constructed a Pearson correlation network for each tissue across all available samples and overlaid treatment‐specific responses as node attributes (Figure 3). The three treatment panels within each tissue share the same underlying correlation‐network topology and differ in the direction and magnitude of treatment‐associated changes. The leaf network showed greater overall connectivity than the root network (Figure 3). Network components in the leaf network comprised features from multiple compound classes, including secondary metabolites, lipids, and peptides/amino acids, indicating that multiple compound classes contributed to the observed co‐variation patterns.

**FIGURE 3:**
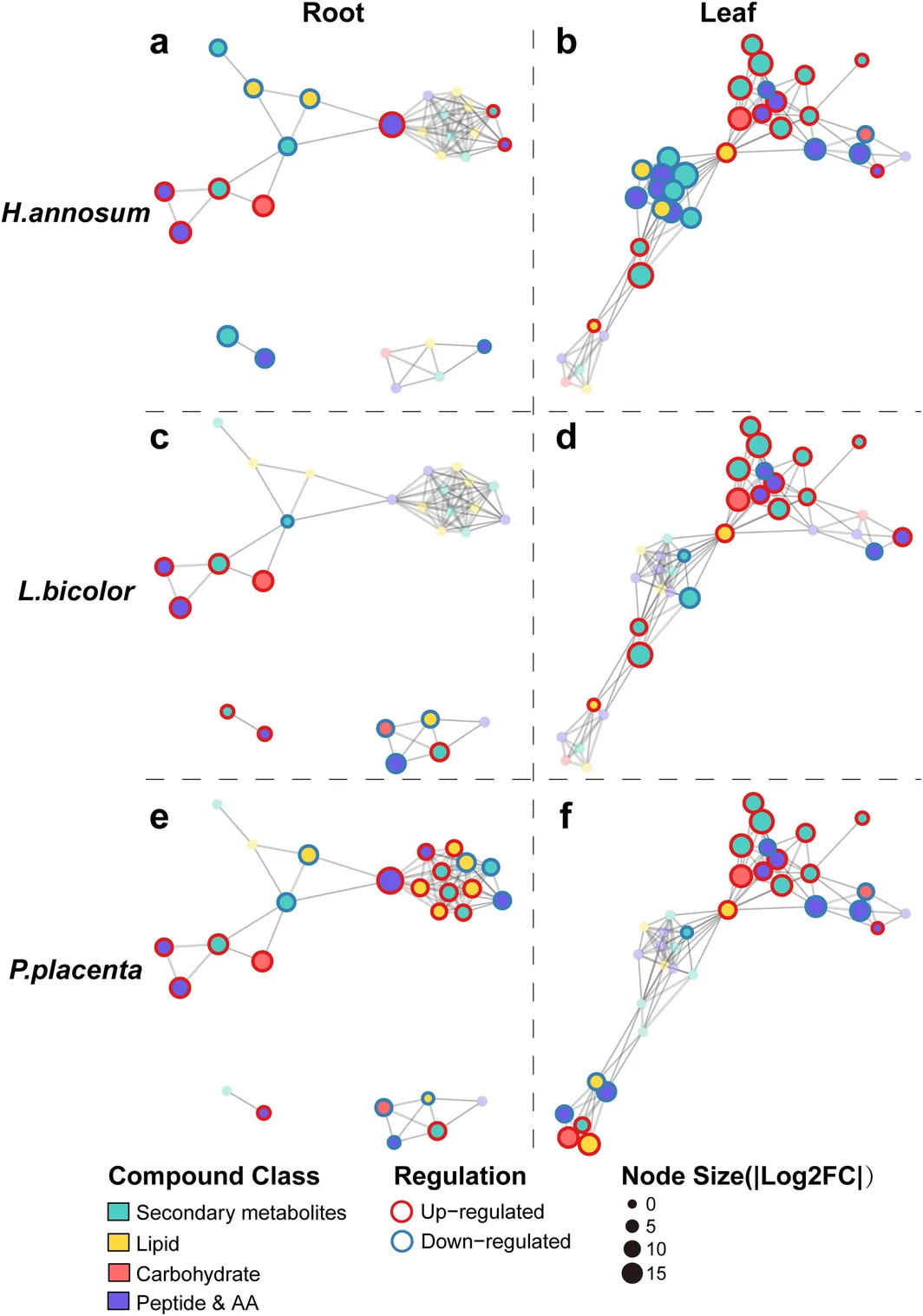
Correlation metabolic networks of high‐confidence differential mass features reveal tissue‐specific metabolic co‐variation following root exposure to fungal VOCs. Pearson correlation networks were constructed within each tissue across all available samples (roots: *n* = 16; leaves: *n* = 15) using the set of high‐confidence differential features (variable importance in projection, VIP > 1, |log_2_FC| > 0.585, FDR‐adjusted *p* < 0.05) as nodes. Edges indicate strong significant Pearson correlations (|r| > 0.7, *p* < 0.05). Root (a, c, e) and leaf (b, d, f) networks are shown, with treatment‐specific overlays for: *H. annosum* (a, b), *L. bicolor* (c, d), and *P. placenta* (e, f). Panels within the same tissue therefore share an identical underlying network topology; only treatment‐associated node attributes differ among panels. Node fill colours denote compound classes based on Multidimensional Stoichiometric Compound Classification (MSCC). Node border colours indicate the direction of regulation for the corresponding treatment relative to control (red, upregulated; blue, downregulated). Node size is proportional to the absolute log_2_ fold change (|log_2_FC|) for the corresponding treatment comparison, with reference sizes shown in the node‐size legend. Network layout was generated using the Fruchterman‐Reingold algorithm.

The root network was sparser overall, while treatment‐specific overlays revealed differences in the direction and magnitude of metabolic responses. In *H. annosum*‐exposed roots (Figure 3a), both up‐ and downregulated features were distributed across the shared root network and included secondary metabolites and peptides/amino acids. In the *L. bicolor* treatment (Figure 3c), comparatively fewer nodes showed large treatment‐associated changes, consistent with the relatively limited number of unique, high‐confidence metabolic changes observed for this treatment. The *P. placenta* overlay (Figure 3e) showed several strongly regulated features within the shared root network, spanning multiple compound classes, including lipids, secondary metabolites, and peptides/amino acids.

Leaf overlays likewise showed treatment‐dependent variation in node‐level responses within the shared network. In the *H. annosum* treatment (Figure 3b), regulated features were distributed across the network and included secondary metabolites and peptides/amino acids. The *L. bicolor* overlay (Figure 3d) showed a broadly similar distribution across the shared network but differed in the magnitude and direction of regulation among individual nodes. The *P. placenta* overlay (Figure 3f) also showed mixed regulation across multiple compound classes, including lipids, secondary metabolites, and peptide/amino‐acid features.

Overall, network components comprised features from multiple biochemical categories. Treatment‐specific differences were therefore reflected primarily in the direction and magnitude of node responses within the shared networks, rather than in differences in network topology or composition. These patterns indicate that fungal VOC exposure is associated with co‐varying metabolic changes across multiple metabolite classes.

### 3.4 Representative Annotated Metabolites Reveal Tissue‐Specific Functional Responses

To clarify the metabolic impact of treatment‐associated changes, annotated high‐confidence differential metabolites representing major functional categories and contrasting treatment‐response patterns were selected for detailed visualisation using dot plots organised by functional group and KEGG pathway classification (Figure 4a,b; Table 1). In roots (Figure 4a), metabolites showed treatment‐specific regulation across compound classes: lipids such as ceramides and phosphatidic acids varied among treatments, multiple tripeptides exhibited upregulation that differed by treatment, and phenolic compounds displayed variable profiles across fungal exposures. In leaves (Figure 4b), salicylic acid 2‐O‐sulfate (SA‐S) showed broad upregulation across all fungal treatments compared to controls. Certain lipids showed *P. placenta* ‐specific accumulation, while rutin was downregulated across all three fungal treatments, with the largest magnitude of decrease observed under *H. annosum*.

**FIGURE 4:**
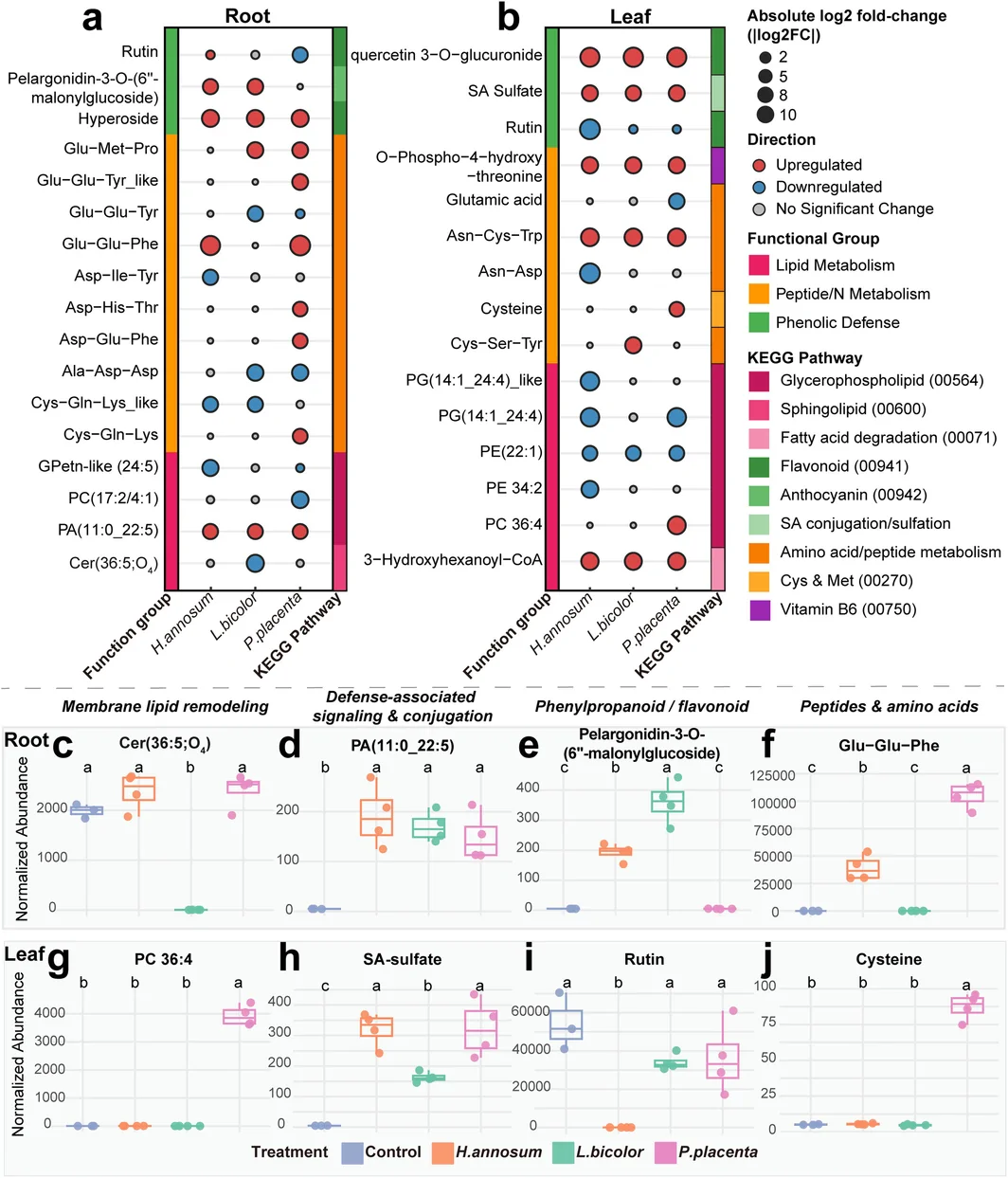
Tissue‐specific metabolite responses to fungal volatile organic compounds (VOCs) in poplar roots and leaves. (a, b) Dot plots summarising responses of selected metabolites in roots (a) and leaves (b) after contact‐free root exposure to VOCs emitted by *H. annosum* (pathogen), *L. bicolor* (mutualist), and *P. placenta* (saprotroph). Dot size represents the absolute log_2_ fold‐change (|log_2_FC|) relative to controls. Red and blue dots indicate significant up‐ and down‐regulation, respectively (FDR‐adjusted *p* < 0.05); grey dots indicate no significant change. Left colour bars denote functional groups; right colour bars indicate KEGG pathway classifications. (c–j) Box plots of median‐normalised abundance for representative metabolites, illustrating four response patterns in roots (c–f) and leaves (g–j): Membrane lipid remodeling (c, g), defence‐associated signalling and conjugation (d, h), phenylpropanoid/flavonoid (e, i), and peptides and amino acids (f, j). Different letters indicate significant differences among treatments (Welch's one‐way ANOVA followed by Games‐Howell post hoc tests, FDR‐adjusted *p* < 0.05; *n* = 4 biological replicates per treatment, except leaf controls *n* = 3). Representative metabolites in panels c‐j were selected from annotated high‐confidence differential features to illustrate major functional categories and contrasting treatment‐response patterns.

**TABLE 1:** Detailed information of representative differential metabolites in poplar roots and leaves responding to fungal VOCs.

Nr	Tissue	Metabolite names	Functional group	KEGG pathway	Functional relevance
1	Root	Cer(36:5;O_4_)	Lipid Metabolism	Sphingolipid metabolism (map00600)	(Li et al. 2022; Liu et al. 2021)
2	Root	PC(17:2/4:1)	Lipid Metabolism	Glycerophospholipid metabolism (map00564)	(Lavell and Benning 2019; Yu et al. 2021)
3	Root	GPetn‐like(24:5)	Lipid Metabolism	Glycerophospholipid metabolism (map00564)	(Lavell and Benning 2019; Yu et al. 2021)
4	Root	PA(11:0_22:5)	Lipid Metabolism	Glycerophospholipid metabolism (map00564)	(Lavell and Benning 2019; Yu et al. 2021)
5	Root	Glu‐Glu‐Phe	Peptide/N metabolism	Amino acid/peptide metabolism	(Agarwal et al. 2025; Hatsugai et al. 2015; Moreau et al. 2005)
6	Root	Asp‐Glu‐Phe	Peptide/N metabolism	Amino acid/peptide metabolism	(Agarwal et al. 2025; Hatsugai et al. 2015; Moreau et al. 2005)
7	Root	Cys‐Gln‐Lys	Peptide/N metabolism	Amino acid/peptide metabolism	(Agarwal et al. 2025; Hatsugai et al. 2015; Moreau et al. 2005)
8	Root	Asp‐Ile‐Tyr	Peptide/N metabolism	Amino acid/peptide metabolism	(Agarwal et al. 2025; Hatsugai et al. 2015; Moreau et al. 2005)
9	Root	Glu‐Glu‐Tyr*	Peptide/N metabolism	Amino acid/peptide metabolism	(Agarwal et al. 2025; Hatsugai et al. 2015; Moreau et al. 2005)
10	Root	Cys‐Gln‐Lys_like	Peptide/N metabolism	Amino acid/peptide metabolism	(Agarwal et al. 2025; Hatsugai et al. 2015; Moreau et al. 2005)
11	Root	Glu‐Met‐Pro	Peptide/N metabolism	Amino acid/peptide metabolism	(Agarwal et al. 2025; Hatsugai et al. 2015; Moreau et al. 2005)
12	Root	Ala‐Asp‐Asp	Peptide/N metabolism	Amino acid/peptide metabolism	(Agarwal et al. 2025; Hatsugai et al. 2015; Moreau et al. 2005)
13	Root	Asp‐His‐Thr	Peptide/N metabolism	Amino acid/peptide metabolism	(Agarwal et al. 2025; Hatsugai et al. 2015; Moreau et al. 2005)
14	Root	Glu‐Glu‐Tyr_like	Peptide/N metabolism	Amino acid/peptide metabolism	(Agarwal et al. 2025; Hatsugai et al. 2015; Moreau et al. 2005)
15	Root	Hyperoside*	Phenolic Defence	Flavonoid biosynthesis (map00941)	(Laffon et al. 2025)
16	Root/leaf	Rutin*	Phenolic Defence	Flavonoid biosynthesis (map00941)	(Kurkin and Kupriyanova 2020; Plett and Martin 2012)
17	Root	Pg‐3‐MalGlc	Phenolic Defence	Anthocyanin biosynthesis (map00942)	(Zhang et al. 2021)
18	Leaf	PE(22:1)	Lipid Metabolism	Glycerophospholipid metabolism (map00564)	(Wewer et al. 2014)
19	Leaf	PG(14:1_24:4)	Lipid Metabolism	Glycerophospholipid metabolism (map00564)	(Lavell and Benning 2019; Yu et al. 2021)
20	Leaf	3‐OH‐C6‐CoA	Lipid Metabolism	Fatty acid degradation (map00071)	(Sun et al. 2017)
21	Leaf	PE(34:2)*	Lipid Metabolism	Glycerophospholipid metabolism (map00564)	(Wewer et al. 2014)
22	Leaf	PC(36:4)*	Lipid Metabolism	Glycerophospholipid metabolism (map00564)	(Wewer et al. 2014)
23	Leaf	PG(14:1_24:4)_like	Lipid Metabolism	Glycerophospholipid metabolism (map00564)	(Lavell and Benning 2019; Yu et al. 2021)
24	Leaf	Cysteine	Peptide/N metabolism	Cysteine and methionine metabolism (map00270)	(Álvarez et al. 2012; Jiang et al. 2024)
25	Leaf	OP4HT	Peptide/N metabolism	Vitamin B6 metabolism (map00750)	(He et al. 2024; Kim and Copley 2012)
26	Leaf	Glutamic Acid*	Peptide/N metabolism	Amino acid/peptide metabolism	(Jiang et al. 2024; Toyota et al. 2018)
27	Leaf	Asn‐Cys‐Trp	Peptide/N metabolism	Amino acid/peptide metabolism	(Agarwal et al. 2025; Hatsugai et al. 2015; Moreau et al. 2005)
28	Leaf	Cys‐Ser‐Tyr	Peptide/N metabolism	Cysteine and methionine metabolism (map00270)	(Agarwal et al. 2025; Hatsugai et al. 2015; Moreau et al. 2005)
29	Leaf	Asn‐Asp	Peptide/N metabolism	Amino acid/peptide metabolism	(Agarwal et al. 2025; Hatsugai et al. 2015; Moreau et al. 2005)
30	Leaf	SA‐S*	Phenolic defence	Salicylate conjugation/sulfation	(Baek et al. 2010; Maruri‐López et al. 2019)
31	Leaf	Q3GA*	Phenolic defence	Flavonoid biosynthesis (map00941)	(Engström et al. 2015)

*Note:* Metabolites marked with an asterisk (*) indicate Level 2 identifications confirmed by MS/MS spectral matching. Unmarked metabolites represent Level 3 (putative) identifications based on MS1 accurate mass only. For tripeptides at Level 3, the indicated amino acid sequences represent one possible arrangement; isomeric sequences cannot be distinguished. Lipid nomenclature: Lipids are annotated as “Class (total carbon atoms: total double bonds)”.

Abbreviations: 3‐OH‐C6‐CoA, 3‐hydroxyhexanoyl‐coenzyme A; GPetn, glycerophosphoethanolamine; OP4HT, O‐phospho‐4‐hydroxy‐L‐threonine; Pg‐3‐MalGlc, pelargonidin 3‐O‐(6″‐O‐malonyl‐glucoside); Q3GA, quercetin‐3‐O‐glucuronide; SA‐S, salicylic acid 2‐O‐sulfate.

Box plot analysis (Figure 4c–j; adj. *p* < 0.05) confirmed distinct response patterns across functional groups. For membrane lipid remodelling, root Cer(36:5;O_4_) was significantly lower in *L. bicolor* ‐exposed plants than in the other three groups (Figure 4c), while leaf PC(36:4) was significantly higher only under *P. placenta* treatment (Figure 4g). For defence‐associated signalling and conjugation, root PA(11:0_22:5) was significantly elevated in all three fungal treatments compared to controls (Figure 4d), while leaf SA‐S similarly showed significant elevation across all fungal treatments (Figure 4h). For phenylpropanoid/flavonoid metabolites, root pelargonidin‐3‐O‐(6″‐malonylglucoside) levels were highest in *L. bicolor* ‐exposed plants and were also significantly elevated in *H. annosum*‐exposed plants compared to controls and *P. placenta* (Figure 4e), while leaf rutin was significantly suppressed only in *H. annosum*‐exposed plants (Figure 4i). For peptides and amino acids, root Glu‐Glu‐Phe levels were highest in *P. placenta* ‐exposed plants, with *H. annosum* also significantly higher than controls and *L. bicolor* (Figure 4f), and leaf cysteine was significantly higher only in *P. placenta* (Figure 4j). Complete response profiles grouped by treatment specificity are provided in Figure S6.

## 4 Discussion

### 4.1 Fungal VOC Exposure Induces Systemic Metabolic Reprogramming

Our results show that exposure to fungal VOCs can induce systemic metabolic reprogramming in *Populus* × *canescens* even in the absence of physical contact. The metabolomics analysis presented here extends our earlier volatile profiling work (Zhu et al. 2026) by examining the downstream metabolic responses of roots and leaves to sustained exposure to VOCs from the three fungi. Using the same pot‐in‐pot exposure system and plant material, we characterised the root and leaf metabolomes after 6 weeks of VOC exposure, providing a complementary view of plant responses at the molecular level. Across the three fungal treatments, poplars showed a shared VOC‐responsive component together with fungus‐ and tissue‐dependent metabolic differences. These findings are consistent with the hypothesis that volatile‐mediated sensing can influence whole‐plant physiology before contact (Rosenkranz et al. 2021). Specifically, *H. annosum* was associated with a pronounced suppression component among high‐confidence metabolic features in leaves but a more balanced pattern of up‐ and downregulation in roots; *P. placenta* induced broad metabolic changes that included selected lipid‐associated responses; and *L. bicolor* produced comparatively few unique high‐confidence metabolic changes (Figure 5). Because each ecological lifestyle was represented by a single fungal species, these contrasting patterns are interpreted here as fungus‐dependent responses rather than as general lifestyle‐specific metabolic programmes.

**FIGURE 5:**
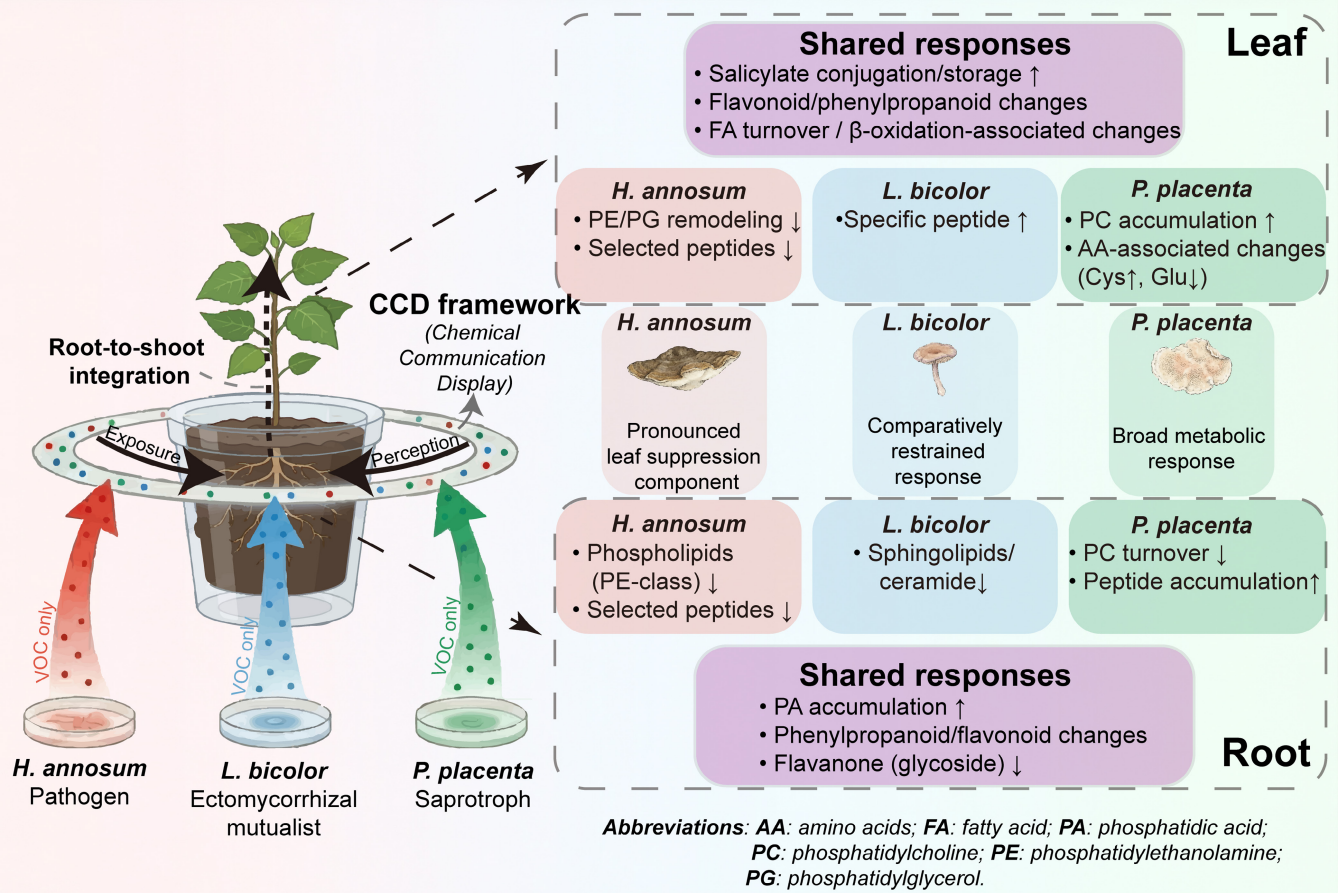
Conceptual model of shared and fungus‐dependent systemic metabolic responses in poplar following belowground exposure to fungal VOCs. The diagram, prepared by the authors using Inkscape, summarizes shared and treatment‐associated metabolic responses in roots and leaves following contact‐free exposure to VOCs from *H. annosum*, *L. bicolor* , and *P. placenta* . The CCD framework is used here as a conceptual framework for interpreting responses to multicomponent fungal VOC environments rather than as evidence for direct decoding of fungal lifestyles.

### 4.2 Chemical Communication Displays (CCDs) as a Framework for Volatile‐Mediated Responses

The differential responses to fungal VOCs can be considered within the framework of chemical communication displays (CCDs) (Figure 5). In the present metabolomic dataset, exposure to VOCs from all three fungi produced a shared metabolic component, while each fungal treatment was also associated with distinct changes in roots and leaves. These treatment‐associated responses involved multiple metabolite classes, including secondary metabolites, lipids, and peptides/amino acids, and the correlation networks likewise revealed co‐varying responses across these biochemical categories. This combination of shared and fungus‐dependent responses is consistent with the idea that plants respond to multicomponent volatile environments rather than to a single universal fungal signal.

CCD theory emphasises that biological meaning can reside in blend composition, ratios, and covariation, rather than in single ‘marker’ compounds (Junker et al. 2018). The previously characterised VOC profiles from the same experiment provide additional chemical exposure context for this interpretation (Zhu et al. 2026). Exploratory GLM and LM analyses linking these VOC profiles (Zhu et al. 2026) to metabolome PC1 scores were consistent with this possibility (Table S1), although these analyses are considered supportive rather than direct evidence for the CCD framework. No individual VOC chemical class consistently predicted metabolome variation across tissues, whereas sesquiterpene class composition showed a significant association with leaf response and treatment identity was associated with both root and leaf metabolome variation. These results are consistent with the possibility that blend‐level VOC composition contributes to differential plant responses. Consistent with this broader framework, Guo et al. (2021) showed that fungal VOC patterns can predict trophic mode and lifestyle across taxa, and quantified phenotypic integration (i.e., compound covariation) within volatilomes. From this perspective, abundant compound classes such as sesquiterpenes may contribute to blend‐level chemical identity rather than necessarily acting as single diagnostic signals. However, the present experiment does not identify the causal VOC components or establish that the observed metabolic differences represent general decoding of fungal lifestyle.

### 4.3 Fungus‐Dependent Responses and Limitations of Lifestyle‐Level Interpretation

Our experimental design included one fungal species representing each ecological lifestyle. Consequently, species identity and ecological lifestyle cannot be disentangled in the present experiment, and the observed metabolic responses are therefore interpreted primarily as fungus‐dependent. Nevertheless, the contrasting ecological strategies of the three fungi provide a relevant framework for considering the observed patterns. This ecological framing is supported by the distinct VOC profiles of the three fungi in the same experiment (Zhu et al. 2026), and by cross‐taxon volatilomics studies reporting associations between fungal ecology and VOC emission patterns (Guo et al. 2021). The metabolic responses observed here also differed among the three fungal treatments in a tissue‐dependent manner. These patterns are therefore consistent with the possibility that fungal ecological strategy contributes to variation in plant VOC responses, but they do not establish general lifestyle‐specific metabolic programmes. Future studies incorporating multiple phylogenetically diverse taxa within each ecological group will be required to test the generality of these patterns.

### 4.4 Shared Metabolic Responses Across Fungal VOCs

Beyond differences among fungal treatments, Grey poplar exhibited a shared VOC‐responsive component, particularly in roots. This suggests that VOC exposure from the three fungi can elicit shared metabolic adjustments. Two metabolites illustrate these shared responses. Phosphatidic acid [PA(11:0_22:5)] accumulated in roots across all three fungal treatments, consistent with its role as an early stress signal linked to MAPK‐mediated signalling (Zhou et al. 2022) and fungal defence responses (Zhao et al. 2013). The accumulation of salicylic acid 2‐O‐sulfate (SA‐S) reflects a broad strategy of salicylate conjugation and mobilisation in response to fungal cues, aimed at balancing immune activation (Baek et al. 2010; Maruri‐López et al. 2019). Within the CCD concept, such shared responses are compatible with plants responding to shared components of fungal VOC mixtures, while fungal‐dependent outcomes may depend on the context and structure of the mixture.

### 4.5 *H. annosum* VOCs Are Associated With Pronounced Leaf Metabolic Suppression

VOC cues from *H. annosum* induced a pronounced metabolic response, particularly in leaves, where the response showed a strong suppression component. This pattern, following prolonged pre‐contact exposure, aligns with previously reported suppression of secondary metabolism during pathogenic interactions, as described for contact‐dependent pathogenicity mechanisms (Shang et al. 2024; Xu et al. 2019). However, the root metabolome showed a more balanced pattern of up‐ and downregulation, indicating that the suppression component response was primarily evident in leaves. Given the six‐week endpoint design, these patterns likely integrate direct VOC‐triggered responses with longer‐term physiological adjustment and should not be interpreted as resolving the temporal sequence of defence signalling.

### 4.6 *P. placenta* VOCs Induce Broad Metabolic Changes With Lipid‐Associated Responses

VOC cues derived from *P. placenta* produced a distinct metabolic response characterised by broad metabolic changes, including lipid‐associated responses alongside shifts in peptide/amino‐acid and secondary‐metabolite features. One reasonable ecological interpretation is that VOC mixtures associated with saprotrophic activity may signal an environmental context linked to substrate turnover, oxidative processes, and microbial competition (Boddy and Hiscox 2016; Mali et al. 2019). In response to such cues, plants may adjust membrane lipid composition to maintain cellular integrity and prepare for potential biotic challenges. Phospholipids such as phosphatidic acid (PA) function both as structural membrane components and as signalling molecules that coordinate stress responses (Zhao et al. 2013; Zhou et al. 2022). The lipid changes observed under *P. placenta* exposure suggest an involvement of membrane lipid remodelling, potentially reflecting plant adjustment to competitive or oxidative conditions associated with saprotrophic activity (Laupheimer et al. 2023). This interpretation should also be considered in the context of ecological flexibility among fungal nutritional strategies, as some saprotrophs can colonise living roots and some mycorrhizal fungi can decompose deadwood (Purahong et al. 2025; Smith et al. 2017). However, because only one saprotrophic species was examined, these responses should be interpreted as specific to *P. placenta* rather than as a general feature of saprotrophic fungi.

### 4.7 *L. bicolor* VOCs Induce Comparatively Restrained Metabolic Reprogramming

VOC cues derived from the mutualist *L. bicolor* elicited comparatively restrained metabolic reprogramming, with relatively few unique high‐confidence changes and substantial overlap with other fungal exposures. Such limited metabolic perturbation is compatible with previous observations that established mutualistic interactions can involve controlled immune modulation rather than strong defence activation (Casarrubia et al. 2016; Kloppholz et al. 2011; Plett et al. 2014). However, whether this relatively restrained response is characteristic of mutualistic fungi more broadly remains to be established. Previous work with *L. bicolor* showed that its VOCs can stimulate lateral root formation in its poplar host even before physical contact occurs (Ditengou et al. 2015). A notable signal at the metabolite level in our dataset was the reduction of the ceramide [Cer(36:5;O_4_)] in *L. bicolor* ‐exposed roots, which may reflect altered sphingolipid‐associated stress signalling. Considering the roles of ceramides in defence and programmed cell death (Li et al. 2022; Liu et al. 2021), this reduction may be compatible with a moderated stress‐associated response and is also consistent with evidence that the *Populus*– *L. bicolor* symbiosis involves fine‐scale jasmonate tuning via effectors such as MiSSP7 (Daguerre et al. 2020; Plett et al. 2014), although such signalling mechanisms were not assessed in the present contact‐free system.

### 4.8 Evidence for Root‐Shoot Integration and Systemic Perception

One striking feature of the system is the strength of root‐shoot integration despite strict belowground exposure. Despite VOC delivery being restricted to the root zone, leaf metabolomes discriminated treatments, suggesting that belowground VOC perception is integrated into the metabolic state of the whole plant. Alongside the paired volatilome results, in which belowground VOC patterns remained sharply distinct while leaf VOC profiles partially converged (Zhu et al. 2026), this supports a spatial organisation in which the roots act as the primary chemosensory interface, exposed to persistent, distinct fungal VOC blends. In contrast, the leaves integrate these cues alongside photosynthetic and whole‐plant demands. Identifying the signalling routes that connect root VOC perception to systemic metabolic outcomes will require time‐resolved sampling and targeted readouts of candidate long‐distance signals.

### 4.9 Mechanistic Implications and Limitations

Mechanistically, responses in the absence of contact imply perception routes beyond classical MAMP (microbe‐associated molecular pattern) recognition (Jones and Dangl 2006). Volatile perception may involve rapid membrane‐associated events, including Ca^2+^ influx, which may couple early sensing to transcriptional and hormonal adjustments (Liu et al. 2025; Zebelo et al. 2012). Furthermore, volatile‐induced Ca^2+^ signatures have been demonstrated by real‐time imaging in Arabidopsis (Aratani et al. 2023). The CCD framework further predicts that plants may respond to blend‐level properties, ratios, and co‐varying modules, rather than to individual compounds, enabling robust discrimination even when single VOCs vary. However, mechanistic inference is constrained by incomplete metabolite annotation (approximately 37%), the relatively limited number of biological replicates, and the chronic six‐week exposure design, which likely integrates direct VOC‐triggered regulation with downstream physiological adjustment. Accordingly, the endpoint metabolome likely integrates early VOC perception with longer‐term physiological acclimation and possible developmental effects, which cannot be temporally distinguished by the present design. The six‐week contact‐free exposure should therefore be regarded as an experimental framework for assessing the effects of prolonged VOC exposure rather than as a fixed natural pre‐contact interval. In addition, because plant growth and morphology were not quantitatively assessed, the observed metabolic changes cannot be directly linked to whole‐plant performance. Unsupervised ordinations identified treatment‐related patterns before supervised modelling. Supervised feature selection and correlation network reconstruction should therefore be regarded as hypothesis‐generating analyses, while the stricter FDR‐filtered differential features provide a more conservative basis for biological interpretation.

## 5 Conclusions: Fungal VOCs Shape Systemic Plant Metabolism

By pairing volatilomics and metabolomics data from the same contact‐free exposure experiment (Zhu et al. 2026), we show that VOCs from contrasting fungal species are associated with systemic metabolic reprogramming in a woody host. *H. annosum*‐associated VOCs were linked to a pronounced suppression component in leaves but a more balanced response in roots, whereas *P. placenta* ‐associated VOCs induced broad metabolic changes that included selected lipid‐associated responses. In contrast, *L. bicolor* ‐associated VOCs produced comparatively few unique high‐confidence metabolic changes. These shared and fungus‐dependent responses are consistent with the chemical communication display (CCD) framework, supporting the idea that belowground fungal VOC chemistry can influence systemic metabolism before physical contact occurs. Whether these response patterns generalise across fungal lifestyles remains to be established.

## Author Contributions

P.Z., M.R., and J.‐P.S. designed research; P.Z. performed research with I.Z. and P.B.S.P., (LC–MS); P.Z. analysed data; P.Z., M.R., A.G., and J.‐P.S. interpreted data. P.Z. wrote the paper with input from all authors.

## Conflicts of Interest

The authors declare no conflicts of interest.

## Supporting information


**Figure S1:** Multivariate visualisation of feature profiles in poplar tissues following root exposure to fungal VOCs. Dimensionality reduction analyses of feature profiles in root (left column) and leaf (right column) tissues using three complementary approaches. (a, b) Principal Component Analysis (PCA) score plots of the preprocessed biological‐sample feature profiles, showing sample distribution along PC1 and PC2, with variance explained indicated on each axis. (c, d) Uniform Manifold Approximation and Projection (UMAP) plots preserving local neighbourhood structure. (e, f) Non‐metric Multidimensional Scaling (NMDS) ordination based on Euclidean distance of scaled data; stress values are indicated. Colours represent treatment groups: Control (blue), *H. annosum* (pink), *L. bicolor* (green), and *P. placenta* (yellow). Sample labels indicate treatment and replicate number.
**Figure S2:** Ranked variable importance in projection (VIP) scores from the OPLS‐DA models of root and leaf metabolomes. Mass features were ranked in descending order according to their VIP scores for root (a) and leaf (b) tissues. Dark green indicates features with VIP > 1, whereas light green indicates features with VIP ≤ 1. The horizontal dashed line indicates the VIP = 1 threshold used for subsequent feature selection.
**Figure S3:** Hierarchical clustering and overlap analysis of discriminant features in poplar tissues following root exposure to fungal VOCs. (a, d) Heatmaps showing *Z*‐score normalised abundance of discriminant features (variable importance in projection, VIP > 1) in root (a) and leaf (d) tissues. Hierarchical clustering (Euclidean distance, complete linkage) was applied to both samples (columns) and features (rows). Colour scale indicates relative abundance (red: elevated; blue: reduced). Top annotation bars indicate treatment groups. Left annotation bars show VIP scores (green gradient) and compound classifications based on Multidimensional Stoichiometric Compound Classification (MSCC). (b, c, e, f) Venn diagrams displaying the overlap of upregulated (log_2_FC > 0.585) (b, e) and downregulated (log_2_FC < −0.585) (c, f) features among treatments in root (b, c) and leaf (e, f) tissues.
**Figure S4:** Volcano plots of discriminant features in poplar tissues following root exposure to fungal VOCs. Volcano plots displaying log_2_(fold change) versus—log_10_ (FDR‐adjusted *p*‐value) for discriminant features (VIP > 1) in root (a–c) and leaf (d–f) tissues. Comparisons are shown for *H. annosum* vs. Control (a, d), *L. bicolor* vs. Control (b, e), and *P. placenta* vs. Control (c, f). Vertical dashed lines indicate fold change thresholds (|log_2_FC| = 0.585); horizontal dashed lines indicate the significance threshold (FDR‐adjusted *p* < 0.05). Red points represent differential features meeting both criteria; grey points indicate features not meeting one or both thresholds. Numbers denote feature identifiers.
**Figure S5:** Van Krevelen diagrams of differential features in poplar tissues following root exposure to fungal VOCs. Van Krevelen plots displaying H/C versus O/C elemental ratios of differential features (VIP > 1, |log_2_FC| > 0.585, FDR‐adjusted *p* < 0.05) in root (a–c) and leaf (d–f) tissues. Panels show responses to *H. annosum* (a, d), *L. bicolor* (b, e), and *P. placenta* (c, f). Node colours indicate compound classifications based on Multidimensional Stoichiometric Compound Classification (MSCC): carbohydrates (pink), lipids (yellow), proteins/peptides/amino acids (purple), secondary metabolites (cyan), and unknown compounds (grey). Node size reflects log_10_‐transformed feature abundance.
**Figure S6:** Complete high‐confidence feature response profiles grouped by treatment specificity. Boxplots displaying normalised abundance of all differentially regulated features (VIP > 1, |log_2_FC| > 0.585, FDR‐adjusted *p* < 0.05) grouped by their response specificity to fungal VOC treatments. H, L, and P represent VOC treatments from *Heterobasidion annosum*, *Laccaria bicolor*, and *Postia placenta*, respectively. (a) General response (regulated by H, L, and P), (b–d) pairwise shared responses (H + L, H + P, L + P), and (e–g) treatment‐specific responses (H‐only, L‐only, P‐only). Colours: Control (Purple blue), *H. annosum* (Orange), *L. bicolor* (Green), *P. placenta* (Pink). Tissue type indicated as Root or Leaf. *n* = 4 biological replicates (*n* = 3 for leaf control). See Figure 4 for representative examples and summary bubble plots.
**Table S1:** GLM and LM analysis of VOC predictors for plant metabolome variation.

## Data Availability

The raw metabolomics data, all processed feature matrices, and the custom R scripts are publicly available in the OSF (Open Science Framework) repository (DOI: 10.17605/OSF.IO/7Y4E8).
