# Supplementary material for: Belowground Fungal Volatile Exposure Induces Systemic Metabolic Reprogramming in Poplar
Source: Physiol Plant. 2026 Oct 9;178(5):e71147. doi: 10.1111/ppl.71147 (PMC13653124; doi:10.1111/ppl.71147)
Supplement: Supplementary file 1 [file PPL-178-e71147-s001.docx]

**Supporting Information for**

**Belowground fungal volatile exposure induces systemic metabolic reprogramming in poplar**

Peiyuan Zhu^1^, Ina Zimmer^1^, Prasath Balaji Sivaprakasam Padmanaban^1^, Maaria Rosenkranz^1,2^, Andrea Ghirardo^1,3^, Jörg-Peter Schnitzler^1,3,4*^

^1^Research Unit Environmental Simulation, Helmholtz Munich, Neuherberg, Germany

^2^Institute of Plant Sciences, Ecology and Conservation Biology, University of Regensburg, Regensburg, Germany

^3^Institute for Translational Metabolism Research, Helmholtz Diabetes Center, Helmholtz Munich / School of Medicine and Health, Technical University of Munich, Munich, Germany

^4^Ecosystem Physiology, Faculty of Environment and Natural Resources, University Freiburg, Germany

^*^**Corresponding author:** Jörg-Peter Schnitzler

**Email:** joergpeter.schnitzler@helmholtz-munich.de

**This PDF file includes:**

Figures S1 to S6

Tables S1

**Supplemental Figures and Tables**

Supplemental figure S1

**Figure S1. Multivariate visualisation of feature profiles in poplar tissues following root exposure to fungal VOCs.**

Dimensionality reduction analyses of feature profiles in root (left column) and leaf (right column) tissues using three complementary approaches. (a, b) Principal Component Analysis (PCA) score plots of the preprocessed biological-sample feature profiles, showing sample distribution along PC1 and PC2, with variance explained indicated on each axis. (c, d) Uniform Manifold Approximation and Projection (UMAP) plots preserving local neighbourhood structure. (e, f) Non-metric Multidimensional Scaling (NMDS) ordination based on Euclidean distance of scaled data; stress values are indicated. Colours represent treatment groups: Control (blue), *H. annosum* (pink), *L. bicolor* (green), and *P. placenta* (yellow). Sample labels indicate treatment and replicate number.

Supplemental figure S2

**Figure S2. Ranked variable importance in projection (VIP) scores from the OPLS-DA models of root and leaf metabolomes.** Mass features were ranked in descending order according to their VIP scores for root (a) and leaf (b) tissues. Dark green indicates features with VIP > 1, whereas light green indicates features with VIP ≤ 1. The horizontal dashed line indicates the VIP = 1 threshold used for subsequent feature selection.

Supplemental figure S3

**Figure S3. Hierarchical clustering and overlap analysis of discriminant features in poplar tissues following root exposure to fungal VOCs.**

(a, d) Heatmaps showing Z-score normalised abundance of discriminant features (variable importance in projection, VIP > 1) in root (a) and leaf (d) tissues. Hierarchical clustering (Euclidean distance, complete linkage) was applied to both samples (columns) and features (rows). Colour scale indicates relative abundance (red: elevated; blue: reduced). Top annotation bars indicate treatment groups. Left annotation bars show VIP scores (green gradient) and compound classifications based on Multidimensional Stoichiometric Compound Classification (MSCC). (b, c, e, f) Venn diagrams displaying the overlap of upregulated (log_2_FC > 0.585) (b, e) and downregulated (log_2_FC < -0.585) (c, f) features among treatments in root (b, c) and leaf (e, f) tissues.

Supplemental figure S4

**Figure S4. Volcano plots of discriminant features in poplar tissues following root exposure to fungal VOCs.**

Volcano plots displaying log_2_(fold change) versus – log_10_ (FDR-adjusted *P*-value) for discriminant features (VIP > 1) in root (a-c) and leaf (d-f) tissues. Comparisons are shown for *H. annosum* vs. Control (a, d), *L. bicolor* vs. Control (b, e), and *P. placenta* vs. Control (c, f). Vertical dashed lines indicate fold change thresholds (|log_2_FC| = 0.585); horizontal dashed lines indicate the significance threshold (FDR-adjusted *P* < 0.05). Red points represent differential features meeting both criteria; grey points indicate features not meeting one or both thresholds. Numbers denote feature identifiers.

Supplemental figure S5

**Figure S5. Van Krevelen diagrams of differential features in poplar tissues following root exposure to fungal VOCs.**

Van Krevelen plots displaying H/C versus O/C elemental ratios of differential features (VIP > 1, |log_2_FC| > 0.585, FDR-adjusted *P* < 0.05) in root (a-c) and leaf (d-f) tissues. Panels show responses to *H. annosum* (a, d), *L. bicolor* (b, e), and *P. placenta* (c, f). Node colours indicate compound classifications based on Multidimensional Stoichiometric Compound Classification (MSCC): carbohydrates (pink), lipids (yellow), proteins/peptides/amino acids (purple), secondary metabolites (cyan), and unknown compounds (grey). Node size reflects log₁₀-transformed feature abundance.

Supplemental figure S6

**Figure S6. Complete high-confidence feature response profiles grouped by treatment specificity.** Boxplots displaying normalised abundance of all differentially regulated features (VIP > 1, |log_2_FC| > 0.585, FDR-adjusted *P* < 0.05) grouped by their response specificity to fungal VOC treatments. H, L, and P represent VOC treatments from *Heterobasidion annosum*, *Laccaria bicolor*, and *Postia placenta*, respectively. (a) General response (regulated by H, L, and P), (b-d) pairwise shared responses (H + L, H + P, L + P), and (e-g) treatment-specific responses (H-only, L-only, P-only). Colours: Control (Purple blue), *H. annosum* (Orange), *L. bicolor* (Green), *P. placenta* (Pink). Tissue type indicated as Root or Leaf. n = 4 biological replicates (n = 3 for leaf control). See Figure 4 for representative examples and summary bubble plots.

**Table S1. GLM and LM analysis of VOC predictors for plant metabolome variation.**

| Term | df | Root PC1_high  χ^2^ (p) | Leaf PC1_high χ^2^ (p) | Root PC1  F (p) | Leaf PC1  F (p) |
| --- | --- | --- | --- | --- | --- |
| Alcohols | 1 | - | 0.31 (0.578) | - | 0.68 (0.425) |
| Aldehydes | 1 | 0.15 (0.703) | 0.04 (0.850) | 0.05 (0.832) | 0.00 (0.953) |
| Alkanes | 1 | 0.23 (0.630) | 0.29 (0.588) | 0.97 (0.342) | 0.47 (0.503) |
| Benzene Derivatives | 1 | 2.42 (0.119) | 0.05 (0.820) | 1.00 (0.334) | 0.25 (0.624) |
| Esters | 1 | - | 0.40 (0.529) | - | 0.68 (0.424) |
| Ketones | 1 | 1.02 (0.311) | 2.89 (0.089) | 0.02 (0.884) | 0.55 (0.473) |
| Monoterpenoids | 1 | - | 0.14 (0.713) | - | 0.03 (0.861) |
| Sesquiterpenoids | 1 | 0.26 (0.612) | 0.12 (0.725) | 0.05 (0.834) | 0.61 (0.448) |
| TotalVOC | 1 | 0.27 (0.606) | 0.35 (0.557) | 0.05 (0.827) | 0.39 (0.541) |
| MT_class | 3 | - | 5.14 (0.162) | - | 0.59 (0.633) |
| SQT_class | 3 | 7.64 (0.054) | **10.68 (0.014)^*^** | 0.68 (0.580) | 1.05 (0.410) |
| Treatment | 3 | **11.09 (0.011)^*^** | 5.14 (0.162) | 2.19 (0.142) | **4.84 (0.022)^*^** |

VOC data for statistical analysis were derived from Zhu et al. (2026). SQT_class and MT_class denote quartile (Q1–Q4) categories of sesquiterpenoid and monoterpenoid *proportions* in total VOC emissions. “–” indicates predictors with zero variance (excluded from the model). Significance: **^*^***P* < 0.05 (ns otherwise).
